# Mapping the overdose crisis in Ontario: geographic disparities in opioid-related harms and services

**DOI:** 10.1186/s12889-025-25103-y

**Published:** 2025-11-13

**Authors:** Farihah Ali, Jordan Mende-Gibson, Sameer Imtiaz, Cayley Russell, Shannon Chellew Paternostro, Sami Aftab Abdul, Nikki Bozinoff, David C. Marsh, Pamela Leece, Jürgen Rehm

**Affiliations:** 1 Institute for Mental Health Policy Research, Centre for Addiction and Mental Health (CAMH), Toronto, M5S 2S1 Canada; 2 Ontario Node, Canadian Research Initiative in Substance Matters (CRISM), Centre for Addiction and Mental Health (CAMH), Toronto, M5S 2S1 Canada; 3 Faculty of Applied Health and Community Studies, School of Applied Health, Clinical Research, Sheridan College, 7899 McLaughlin Rd, Brampton, ON L6Y 5H9 Canada; 4 Campbell Family Mental Health Research Institute, Centre for Addiction and Mental Health, 250 College St, Toronto, ON M5T 1R8 Canada; 5 Department of Family and Community Medicine, University of Toronto, 500 University Avenue, 5th floor, Toronto, M5G1V7 Canada; 6 Dalla Lana School of Public Health, University of Toronto, 480 University Ave, Suite 300, Toronto, ON M5G 1V2 Canada; 7 School of Epidemiology and Public Health, Faculty of Medicine, University of Ottawa, 600 Peter Morand Crescent, Ottawa, Ottawa, K1G 5Z3 Ontario, Ontario Canada; 8 Northern Ontario School of Medicine University, 935 Ramsey Lake Rd, Sudbury, ON P3E 2C6 Canada; 9 Health Science North Research Institute, 56 Walford Rd, Greater Sudbury, ON P3E 2H3 Canada; 10 ICES North c/o HSNRI Research Services, 56 Walford Road, Sudbury, ON P3E 2H2 Canada; 11 Public Health Ontario, 661 University Avenue, Suite 1701, Toronto, ON M5G 1M1 Canada; 12 Department of Psychiatry, Faculty of Medicine, University of Toronto, 250 College Street, 8th floor, Toronto, ON M5T 1R8 Canada; 13 Faculty of Medicine, Institute of Medical Science, University of Toronto, Medical Sciences Building, 1 King’s College Circle, Room 2374, Toronto, ON M5S 1A8 Canada; 14 Center for Interdisciplinary Addiction Research (ZIS), Department of Psychiatry and Psychotherapy, University Medical Center Hamburg- Eppendorf (UKE), Martinistraße 52, Hamburg, 20246 Germany; 15 Program on Substance Abuse and WHO European Region Collaboration Centre, Public Health Agency of Catalonia, Aragó Street 330, Barcelona, Catalonia 08009 Spain; 16 250 College St, Toronto, ON M5T 1R8 Canada

**Keywords:** Overdose crisis, Rurality, Northern Ontario, Harm reduction, Opioid agonist treatment, Opioid-Related harms

## Abstract

**Background:**

Opioid-related harms and deaths remain a persistent public health crisis across Ontario, Canada, with non-urban regions facing a disproportionate burden. However, discussions of opioid-related harms across Ontario’s geographic regions have provided an oversimplified assessment, contrasting rural and urban regions which mask the unique challenges and true disparities faced by sparsely populated communities, which are commonly located in the Northern regions. Our study aims to provide a more in depth understanding of the opioid crisis in Ontario across different geographic classifications in accordance to population size, such as rural, urban, and sparsely populated regions, presenting data in both absolute numbers and crude rates with contextual grounding of regional characteristics. A number of different opioid-related indicators such as hospitalizations, overdose rates, opioid service provision and harm reduction supply distribution were analyzed across all 34 of Ontario’s public health units (PHUs) to understand the differences in these indicators based on region across the province. The findings can inform the development of targeted interventions and improve service accessibility for those most affected by the overdose crisis in Ontario.

**Methods:**

Publicly-available secondary data for each PHU was collected from several provincial and national data sources and analyzed between November 2024 and January 2025. Annual data from 2022 to 2023 on opioid-related harms, opioid agonist treatment (OAT) prescribers and engagement, and the distribution of harm reduction supplies, as well as annual data from 2024 on opioid-inclusive service provision, were collected. Using Statistics Canada’s 2023 Health Region Peer Group Classification, the PHUS were grouped into four geographic classifications: sparsely populated, rural, urban/rural mix, and urban. Crude average rates were calculated for all indicators. Statistical analysis was performed to assess significance of indicators between regions.

**Results:**

Sparsely populated PHUs were primarily located in Northern Ontario, while rural, urban/rural mix, and urban PHUs were mainly concentrated in Southern Ontario. Urban PHUs have the highest number and lowest rate of opioid-related harms (e.g. 947 opioid-related deaths, representing a rate of 12.5 per 100,000 population), while sparsely populated PHUs reflect the opposite trend (e.g. 158 opioid-related deaths, representing a rate of 44.2 per 100,000 population). A similar pattern emerges for harm reduction services and naloxone distribution. The number of treatment services is highest in rural PHUs (*n* = 237) and lowest in sparsely populated PHUs (*n* = 83), despite having the highest rate. OAT prescribers, OAT engagement, and needle distribution follow a similar trend. Statistical significance was found between geographic regions for most indicators, except opioid-inclusive support services, harm reduction services, and naloxone distribution.

**Conclusion:**

Sparsely populated and rural PHUs experience the highest burden of opioid-related harms, coupled with limitations in service accessibility, demonstrating a clear need for additional harm reduction services. Decision-makers may be misled into underestimating the crisis in non-urban areas as a result of oversimplified reporting, resulting in inadequate support for these regions. Addressing these disparities is key to reducing opioid-related mortality and ensuring equitable access to life-saving services across Ontario.

**Supplementary Information:**

The online version contains supplementary material available at 10.1186/s12889-025-25103-y.

## Background

Opioid-related harms and deaths continue to represent a national crisis, with 8,606 deaths recorded in 2023, the highest ever reported [1]. In Ontario, the highest number of opioid-related deaths was recorded in 2021, with a total of 2,924 deaths in 2021 [2–5]. However, this growing crisis is not experienced equally across the province. Evidence indicates that rural and non-urban communities face a disproportionately higher burden of opioid-related harms, including higher rates of emergency department (ED) visits, hospitalizations, and deaths [6–8]. These disparities are closely linked to gaps in harm reduction and other services for people who use opioids, such as safer supply programming, mobile outreach supports, and supervised consumption services, with evidence highlighting the ability of these programs to reduce opioid-related morbidity and mortality [9–11]. 

Ontario’s health care system is overseen by the Ministry of Health, with programming delivered regionally through 34 Public Health Units (PHUs), supported by a network of 58 Ontario Health Teams that provide integrated health care and public health services to local communities [12, 13]. However, PHUs vary significantly in geographic size, population density, and healthcare infrastructure, which all have major implications for service provision and accessibility. Discussions around opioid-related harms and service availability across geographic regions in Ontario frequently contrast rural and urban regions, highlighting their stark differences [14–16]. However, this binary classification oversimplifies the geographic diversity across the province, overlooking the unique challenges across other regions [14]. For instance, communities in Northern Ontario, characterized by very low population densities, are often grouped with broader rural regions in data reporting. However, most rural regions generally have higher population densities and greater service availability compared to these sparsely populated Northern communities [14, 17, 18]. This broad classification blurs important differences, making it hard to identify unique challenges faced by Northern communities and to develop targeted interventions that address their specific needs and barriers to care [14]. For example, the Rural Ontario Institute classifies Northern regions (excluding Indigenous communities) as rural, reinforcing a generalized definition of rurality that does not account for population differences or geographic remoteness [16]. 

Another challenge in understanding the true extent of opioid-related harms and service provision across the province lies in how data is interpreted. While many data sources report both absolute numbers and crude rates for opioid-related harms, these figures must be understood in tandem with regional characteristics, such as population size, density, and service accessibility, to provide an accurate picture of need [19]. Relying on either metric alone, or looking solely at opioid-related harms across the province, without this contextual grounding, can lead to misinterpretations and does not provide a comprehensive understanding of the opioid crisis in Ontario. Further highlighting the importance of this research, the Chief Medical Officer of Health (CMOH) of Ontario’s 2023 Annual Report discussed the need for such comprehensive data on opioid-use and harms, as well as the need for continued monitoring and reporting of opioid-inclusive programs in Ontario–programs that offer integrated supports or services for individuals who use opioids (e.g. opioid agonist therapy [OAT] service, rapid access addiction medicine [RAAM] clinics, addiction supportive housing [ASH], peer-led support groups) [4]. 

Our study aims to provide a more concrete understanding of the opioid crisis in Ontario, presenting data in both absolute numbers and crude rates, ensuring a more balanced and accurate assessment. The study analyzes opioid-related harms, opioid-inclusive service provision, OAT prescribers and engagement, and the distribution of harm reduction supplies (needle and naloxone distribution) across all 34 of Ontario’s PHUs, categorized into four geographic classifications based on Statistics Canada’s Health Region Peer Group Classifications: [20] urban, urban/rural mix, rural, and sparsely populated. Given that opioid-related harms, opioid-inclusive services, and the distribution of harm reduction supplies vary widely across different regions, population sizes of regions were accounted for to ensure meaningful comparisons. By analyzing these data broken down by geographic region, we hope to identify high-need regions which can better inform policy and decision making regarding service needs. Further, by collecting data on OAT prescribers and engagement across the geographic regions, in addition to opioid-inclusive service provision, this study aims to develop a more thorough understanding of the landscape of OAT service provision, which is the preferred first line treatment for opioid use disorder in Canada; reducing the risks of overdose by managing cravings, maintaining tolerance, and preventing withdrawals [4, 21]. These findings can provide a clearer understanding of where resources are most urgently needed, helping to inform targeted interventions and improve service accessibility for those most affected by the overdose crisis in Ontario.

## Methods

### Study design, data sources, and indicators

This study drew on publicly-available secondary data from several data sources, which were collected and analyzed between November 2024 and January 2025. See Table 1 for a description of data sources and indicators.

**Table 1:** Data Sources, Indicators, Descriptions, and definitions

Data Source	Indicators and Associated Year	Description	Definitions
Public Health Ontario (PHO) *Substance Use and Harms Tool* [5, 22]	Opioid-Related ED Visits (2022–2023) Opioid-Related Hospitalizations (2022–2023) Opioid-Related Deaths (2022–2023) Population Size (2022–2024)	PHO compiles data from multiple sources, including the National Ambulatory Care Reporting System (ED visits), the Discharge Abstract Database (for hospitalizations), and the Ontario Substance-related Death Database from the Office of the Chief Coroner for Ontario for opioid-related deaths [5].	*ED Visits and Hospitalizations*: Cases of opioid poisonings where specific opioids were confirmed (opium, heroin, codeine and derivatives, morphine, hydromorphone, oxycodone, methadone, fentanyl and derivatives, tramadol, and other opioids, synthetic and unspecified narcotics; see PHO’s *“Technical Notes: Substance Use and Harms Tool*for ICD-10-CA Codes) [5, 12]. *Opioid-Related Deaths*: Confirmed and probable cases involving opioids (codeine, fentanyl and analogues, heroin, hydrocodone, hydromorphone, methadone, morphine, nitazenes, and oxycodone). Importantly, opioid toxicity was considered as a contributor to the cause of death in these cases [12].
Ontario Drug Policy Research Network (ODPRN) *Ontario Opioid Indicator Too* [23]	OAT Provision, including: OAT Prescribers (2022–2023) OAT Engagement (2022–2023) Distribution of Harm Reduction Supplies Naloxone Distribution (2022–2023) Needle Distribution (2022–2023)	Number of OAT prescribers and OAT clients (referred to as OAT engagement in this analysis), sourced from the Narcotics Monitoring System (NMS) [24]. Naloxone dose distribution data was sourced from the Ontario Drug Benefit (ODB) database. Data on needle distribution were sourced from the Ontario Ministry of Health.	*OAT Prescribers*: Unique providers who prescribed methadone, buprenorphine, or slow release oral morphine (SROM). Note: The prescriber’s location is determined based on the client’s residence, meaning a single prescriber may be counted in multiple PHUs if they provide care to clients across different regions through virtual care [24]. *OAT Engagement*: Both new and existing clients dispensed OAT medications [23]. The new clients dispensed OAT medications reflected those who were not dispensed a prescription opioid for OAT in a predefined period prior to their first prescription, compared to existing OAT clients who had prior OAT prescriptions [23]. The predefined period was informed by prescriber consultations, and by the type of OAT–shorter periods for more regularly dispensed forms, like methadone (30 days); or longer periods for less frequently dispensed and longer-acting forms, like implantable buprenorphine (270 days) [24]. *Naloxone Distribution*: Includes both community- and pharmacy- provided naloxone doses. Each naloxone kit includes two naloxone doses. Community-provided naloxone doses are distributed by community-based organizations and initiatives, excluding services such as police, fire, and St. John Ambulance.^(24)^ *Needle Distribution*: Total needles distributed through the Needle Exchange Program (NEP).^(24)^
ConnexOntario [25]	Opioid-inclusive services (treatment services, support services, harm reduction, counselling services) Reflective as of January 2025	Opioid-inclusive services extracted from the ConnexOntario Health Services Information Database [26].	*Opioid-Inclusive Harm Reduction Services*: Any program that provides harm reduction services for opioid use. *Opioid-Inclusive Treatment Services*: Bed-based treatment, bed-based supportive treatment, medication-assisted treatment, intensive/day treatment, community-based withdrawal management, RAAM clinics, medical inpatient withdrawal management, crisis management and OAT programs. *Opioid-Inclusive Support Services*: ASH, case management, peer/self-help, and support within housing. *Opioid-Inclusive Counselling Services*: Individual and group counselling, and family support.
Health Canada Website [27, 28]	Safer Supply Programs (SSPs), Consumption and Treatment Services/Supervised Consumption Sites (CTS/SCS), Drug Checking Services Reflective as of January 2025	Supplemental harm reduction service data on SSPs, CTS/SCS, and Drug Checking Services [27, 28].	*Harm Reduction*: CTS/SCS, SSPs, drug checking services, and mobile outreach.
Ontario Government Website [29]	Harm Reduction Mobile Services Reflective as of January 2025	Data on mobile services that distribute harm reduction supplies [29].	

### Data extraction and organization

To ensure consistency in the data collection process, all indicators were gathered at the PHU level. Both PHO [22] and ODPRN [23] provided data that was already disaggregated by PHU.

We utilized Statistics Canada’s 2023 Health Region Peer Group Classification to classify PHUs into geographic regions. Statistics Canada has grouped all national health units in Canada based on shared socio-economic and demographic factors, including population density (measured in people per km^2^). Each grouping has also been assigned a population density rating of “very low”, “low”, “medium”, “high”, or “very high” to facilitate comparisons across regions.

For this study, PHUs were reclassified into four broader geographic categories, using Statistics Canada’s Peer Group classification as a guide. Our classifications, with the population density ranges from Statistics Canada [20], were as follows:

1. Sparsely populated PHUs (low density): 0.04 to 7.6 people per km^2^
2. Rural PHUs (medium density): 2 to 258 people per km^2^
3. Urban/rural mix (high density): 5.7 to 1,781 people per km^2^
4. Urban PHUs (very high density): 42.7 to 5,186 people per km^2^

Since the Peer Group classification of Statistics Canada incorporated factors beyond population density (mainly socio-economic conditions), some overlap existed in the density ranges among geographic categories. This classification system allowed for more precise comparisons of opioid-related harms and service availability across Ontario’s regions.

For opioid-inclusive service data sourced from ConnexOntario [25], by which data were organized by counties, regions and municipalities, additional steps were taken to ensure alignment with the appropriate PHU. These data were aggregated and matched to PHU boundaries using a searchable database by the Association of Local Public Health Agencies (alPHa) that linked municipalities to their corresponding PHUs [30]. Similarly, harm reduction service data sourced from Health Canada and the Ontario government were organized by cities, which were also matched to PHU boundaries using the alPHa database [30]. 

Additionally, ConnexOntario categorized services based on their primary function, while also documenting any additional services they offered. Services were categorized and mapped according to the primary service type identified in the ConnexOntario Health Services Information Database. Relevant opioid-inclusive services were categorized into four main groups, as organized by ConnexOntario: Opioid-Inclusive Treatment Services (which included bed-based treatments, medication assisted treatments [such as OAT], withdrawal management programs, and intensive/day treatments), Support Services (which included case management, ASH, and family support), Harm Reduction Services (identified generally as “harm reduction”), and Counselling Services (identified as “counselling and treatment”). Any services that fell outside these classifications were excluded from this analysis. Further, data on harm reduction services retrieved from Health Canada’s website and the Ontario government’s website were cross referenced with the ConnexOntario database to ensure there were no duplications of reported programs.

All spreadsheets created by the research team for the purpose of this analysis included detailed metadata, such as data sources, collection dates, and any transformations applied to standardize values across datasets for accurate reporting and data management.

### Data analysis

#### Descriptive analysis

Population data for 2022–2024 were collected for each PHU. 2022 and 2023 population data were used to calculate crude average rates for opioid-related harm indicators (ED visits, hospitalizations, and deaths), as these were the most complete datasets available at the time of data collection. In contrast, 2024 population data were used to calculate crude average rates for opioid-inclusive services, as it was the most recent population estimate available.

Crude rates were estimated for individual PHUs, as well as by geographic region (rural, urban, urban/rural mix, and sparsely populated), and overall for Ontario. To generate crude rates, first, each indicator was summed to get the absolute numbers for each year. Then, crude rates for 2022 and 2023 individually were calculated by dividing the indicator with the corresponding population data (ex. ED visits from 2022 were divided by 2022 population data), and then multiplied by 100,000 to get crude rates per 100,000 population, which were then averaged across the two data years. For service availability, the number of services were instead divided by the 2024 population for each region.

Throughout the paper, crude rates are presented descriptively, with both crude rates and absolute numbers presented for each indicator in the form of a graph.

### Statistical analysis

To determine if the rates were significantly different across geographical regions, one-way analysis of variance (ANOVA) was performed, followed by Tukey’s honestly significant difference (HSD) post hoc test if the main effect was significant. Model assumptions were examined via diagnostic plots (i.e., Q-Q plots, Histogram of Residuals, Residuals vs. Fitted Plot, and Residuals vs. Square Root of Standardized Residuals) and statistical tests (i.e., Shapiro Wilk Test, Levene’s Test, and Cook’s Distance). If homoscedasticity was violated, Welch’s ANOVA was used instead, followed by a Games-Howell post hoc test. If normality was violated, the Kruskal-Wallis test was used instead, followed by Dunn’s post hoc with Benjamini-Hochberg correction. A *p*-value of < 0.05 was considered statistically significant. All analyses were performed using RStudio (Version 2024.12.0 + 467).

### Ethics approval

No ethics approval was required for this study, as it exclusively utilized publicly-available secondary data.

## Results

### Geographical distribution of ontario PHUs

Ontario’s 34 PHUs were categorized based on population density as rural, urban, urban/rural mix, or sparsely populated. Of these, 19 PHUs (56%) were rural, six PHUs (18%) were urban/rural mix, five PHUs (15%) were urban, and four PHUs (12%) were sparsely populated.

**Fig. 1:**
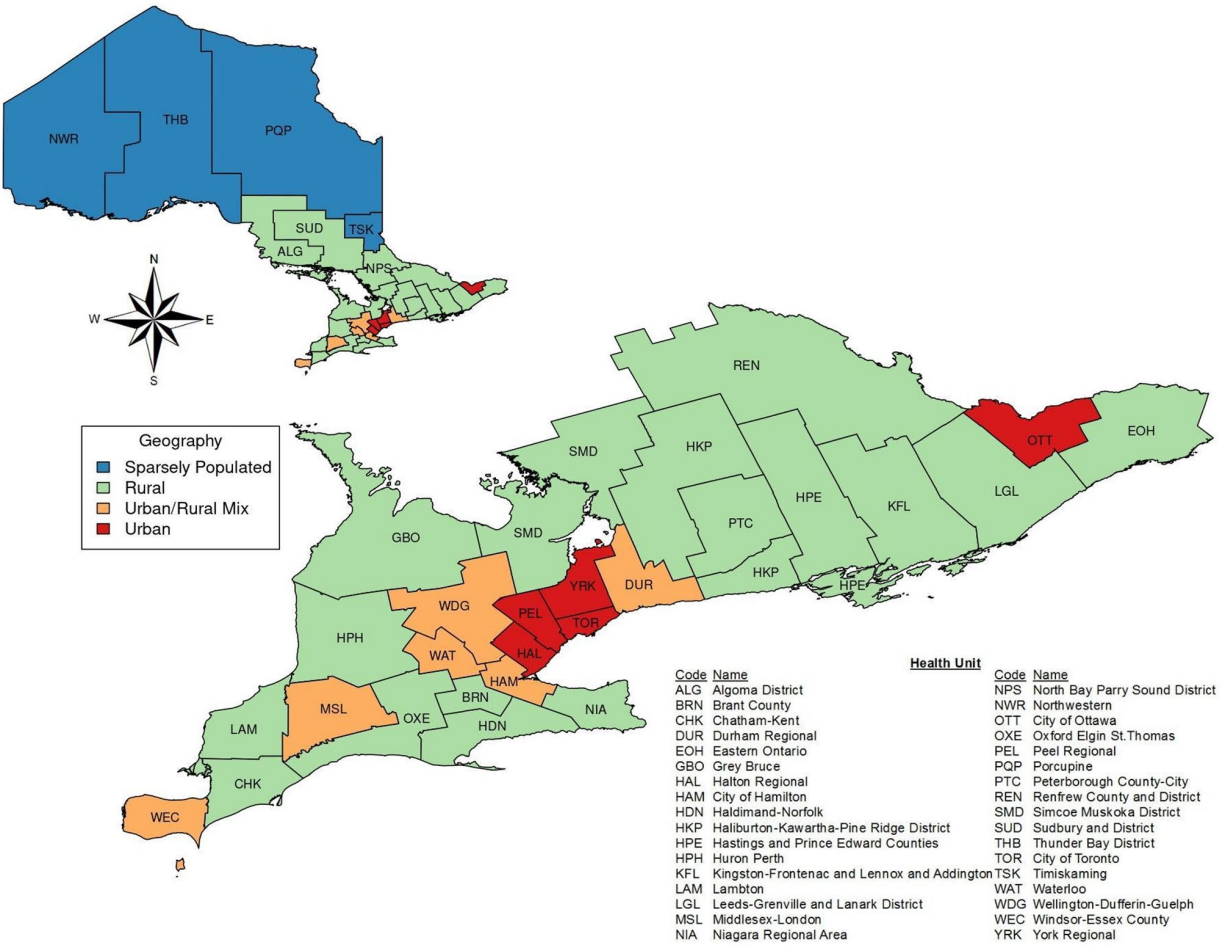
Geographical Distribution of Ontario PHUs

The sparsely populated PHUs were primarily located in Northern Ontario, covering vast geographic areas with low population densities. The rural PHUs were distributed across Southern Ontario and parts of North East Ontario, with a concentration in central and eastern parts of the province. These PHUs were comprised of smaller towns and regions with moderate population densities. Urban/rural mix PHUs were situated in regions where urban centers were surrounded by rural areas, blending both environments. These PHUs were typically located around major cities in Southern Ontario and had high population densities. Lastly, the urban PHUs were concentrated in Southern Ontario, particularly within the Greater Toronto Area and other major metropolitan regions. These PHUs had very high population densities. Figure 1 illustrates the geographical distribution of Ontario’s PHUs.

### Opioid-related harms and service availability indicators

#### Opioid-related harms

##### ED visits

**Fig. 2:**
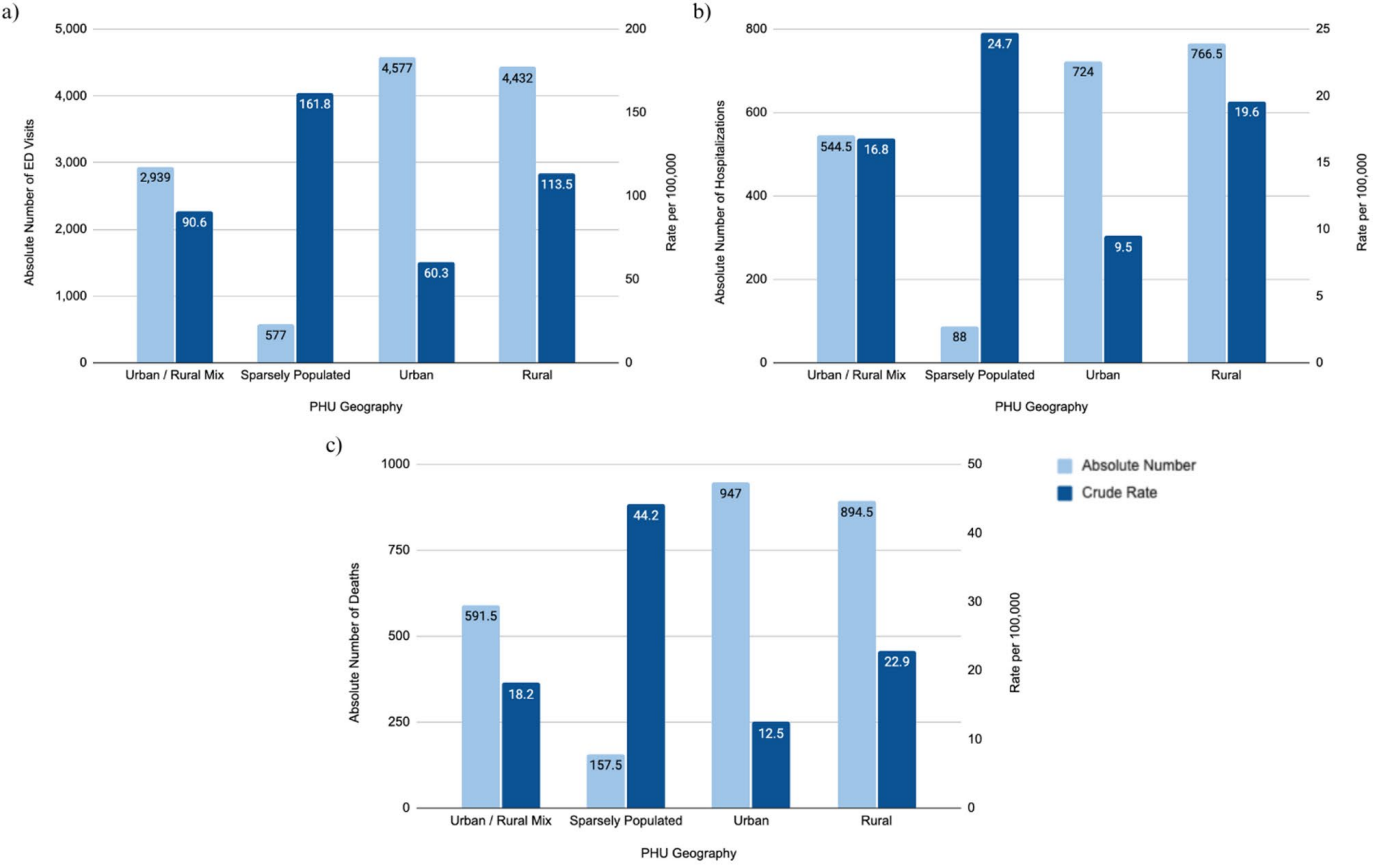
The 2022-2023 average number and rate of opioid-related harms in urban, urban/rural mix, rural, and sparsely populated regions in Ontario. The following indicators are presented: (**a**) ED visits, (**b**) Hospitalizations, and (**c**) Deaths

Between 2022 and 2023, the average number of opioid-related ED visits was 12,525 with a crude rate of 83.0 per 100,000 population for all of Ontario. Across all regions, sparsely populated PHUs reported the highest average rate of opioid-related ED visits (161.8 per 100,000 population), followed by rural and urban/rural mix PHUs (113.5 and 90.6 per 100,000 population, respectively). Conversely, urban PHUs had the lowest rate (60.3 per 100,000 population). See Fig. 2(a) which highlight**s** absolute numbers and crude rates of opioid-related ED visits.

Opioid-related ED visit rates were significantly different across rurality as assessed by omnibus test (Appendix A, Table 1). Pairwise comparisons suggest sparsely populated PHUs had a significantly higher average rate compared to urban PHUs (sparsely populated vs. urban: *p* = 0.0316). No significant differences were found between other geographic classifications.

##### Hospitalizations

Between 2022 and 2023, the average number of opioid-related hospitalizations was 2,123 with a crude rate of 14.1 per 100,000 population for all of Ontario. Sparsely populated PHUs reported the highest average rate of opioid-related hospitalizations (24.7 per 100,000 population), followed by rural PHUs (19.6 per 100,000 population), and urban/rural mix PHUs (16.8 per 100,000 population). Urban PHUs reported the lowest rate (9.5 per 100,000 population). See Fig. 2(b) for absolute numbers and crude rates of opioid-related hospitalizations.

Hospitalization rates were significantly different across rurality as assessed by omnibus test (Appendix A, Table 2). Rural and sparsely populated PHUs had significantly higher rates of opioid-related hospitalizations compared to urban PHUs (urban vs. rural: *p* = 0.0064; urban vs. sparsely populated: *p* = 0.0015). No significant differences were found between other geographical classifications.

##### Opioid-related deaths

Between 2022 and 2023, the average number of opioid-related deaths was 2,591 with a crude rate of 17.2 per 100,000 population for all of Ontario. Sparsely populated PHUs recorded the highest average rate of opioid-related deaths (44.2 per 100,000 population), followed by rural PHUs (22.9 per 100,000 population), urban/rural mix PHUs (18.2 per 100,000 population), then urban PHUs (12.5 per 100,000 population). See Fig. 2 (c) for absolute number and crude rates of opioid-related deaths.

Opioid-related deaths were significantly different across rurality as assessed by omnibus test (Appendix A, Table 3). Sparsely populated PHUs had significantly higher rates of opioid-related deaths compared to urban and urban/rural mix PHUs (sparsely populated vs. urban: *p* = 0.0027 and sparsely populated vs. urban/rural mix: *p* = 0.032). No other significant differences were observed.

#### Opioid-inclusive programs

There were 1,581 total opioid-inclusive programs across Ontario as of January 2025 representing a rate of 10.2 per 100,000 population. Service distribution varied across geographic classifications.

##### Treatment programs

**Fig. 3:**
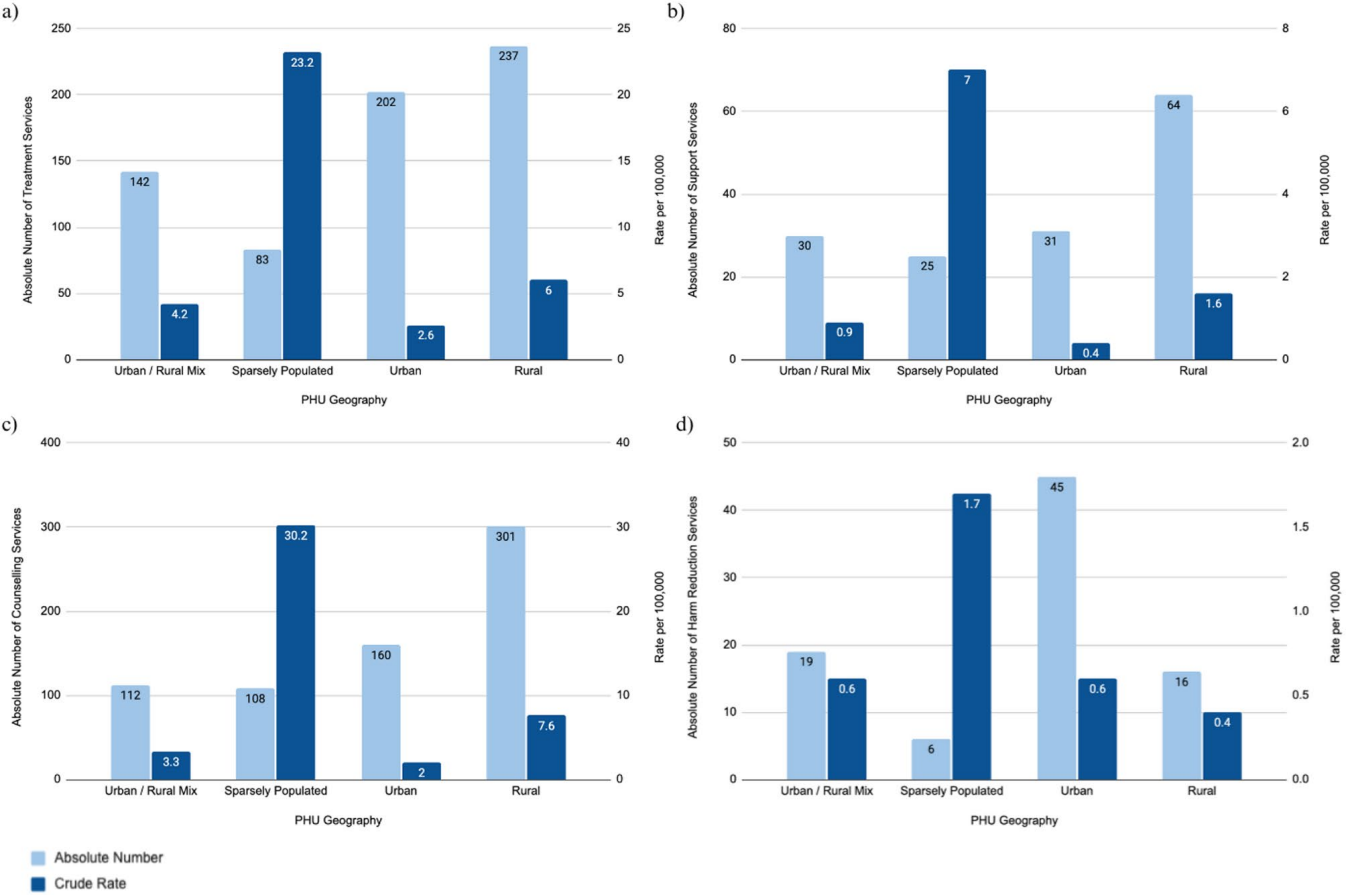
The number and rate of opioid-inclusive services in urban, urban/rural mix, rural, and sparsely populated regions in Ontario. The following indicators are presented: (**a**) Treatment Services, (**b**) Support Services, (**c**) Counselling Services, and (**d**) Harm Reduction Services

There were 664 opioid-inclusive treatment programs across Ontario as of January 2025, representing a rate of 4.3 per 100,000 population. Sparsely populated PHUs had the highest rate of treatment programs (23.2 per 100,000 population), followed by rural PHUs (6.0 per 100,000 population), urban/rural mix PHUs (4.2 per 100,000 population), then urban PHUs (2.6 per 100,000 population). See Fig. 3 (a) which highlight**s** absolute numbers and crude rates of opioid-inclusive treatment services.

The availability of treatment programs was significantly different across rurality as assessed by omnibus test (Appendix A, Table 6). Sparsely populated PHUs had significantly higher rates of opioid-inclusive treatment programs compared to urban, urban/rural mix, and rural PHUs (sparsely populated vs. urban: *p* = 0.0004, sparsely populated vs. urban/rural mix: *p* = 0.0136, and sparsely populated vs. rural *p* = 0.0194). Rural PHUs were also significantly higher than urban PHUs (urban vs. rural: *p* = 0.0185).

##### Support programs

There were a total of 150 support programs across Ontario as of January 2025, representing a rate of 1.0 per 100,000 population. Sparsely populated PHUs reported the highest rate of support programs (7.0 per 100,000 population), followed by rural PHUs (1.6 per 100,000 population), urban/rural mix PHUs (0.9 per 100,000 population), then urban PHUs (0.4 per 100,000 population). See Fig. 3 (b) which highlight**s** absolute numbers and crude rates of opioid-inclusive support services.

There were no significant differences found in the rate of support services between geographic regions (Appendix A, Table 7).

##### Counselling programs

There were a total of 681 counselling programs across Ontario as of January 2025, representing a rate of 4.4 per 100,000 population. Sparsely populated PHUs reported the highest rate of counselling programs (30.2 per 100,000 population), followed by rural PHUs (7.6 per 100,000 population), urban/rural mix PHUs (3.3 per 100,000 population), then urban PHUs (2.0 per 100,000 population). See Fig. 3 (c) which highlight**s** absolute numbers and crude rates of opioid-inclusive counselling services.

The rate of counselling programs was significantly different across rurality as assessed by omnibus test (Appendix A, Table 9). The rate of counselling programs in urban PHUs were significantly lower than rural and sparsely populated PHUs (urban vs. rural: *p* = 0.014 and urban vs. sparsely populated *p* = 0.0009). Urban/rural mix PHUs were also significantly lower than rural and sparsely populated PHUs (urban/rural mix vs. rural: *p* = 0.0366 and urban/rural mix vs. sparsely populated *p* = 0.0018). Rural PHUs reported significantly lower rates than sparsely populated PHUs (rural vs. sparsely populated: *p* = 0.0366).

##### Harm reduction services

There were a total of 86 harm reduction programs across Ontario as of January 2025, representing a rate of 0.6 per 100,000 population. Sparsely populated PHUs had the highest rate of harm reduction programs (1.7 programs per 100,000 population), followed by urban/rural mix PHUs (0.6 per 100,000 population) and urban PHUs (0.6 per 100,000 population), then rural PHUs (0.4 per 100,000 population). See Fig. 3 (d) which highlight**s** absolute numbers and crude rates of opioid-inclusive harm reduction services.

There were no significant differences found in the rate of harm reduction services between geographic regions (Appendix A, Table 8).

#### OAT provision

##### OAT prescribers

**Fig. 4:**
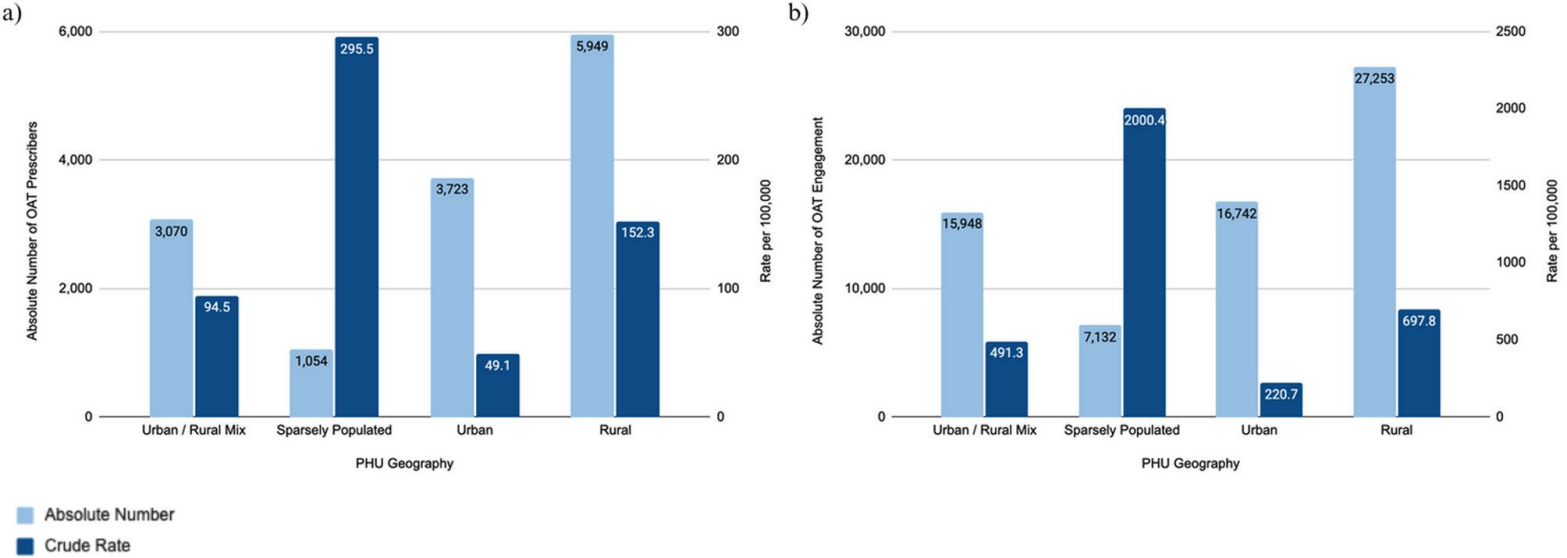
The 2022-2023 average number and rate of OAT prescribers and engagement in urban, urban/rural mix, rural, and sparsely populated regions in Ontario. The following indicators are presented: (**a**) OAT Prescribers, and (**b**) OAT Engagement

Between 2022 and 2023, the average number of OAT prescribers was 13,795 with a crude rate of 91.4 per 100,000 population for all of Ontario. Sparsely populated PHUs reported the highest average rate of OAT prescribers (295.5 per 100,000 population). Rural PHUs also reported higher-than-average rates (152.3 per 100,000 population), followed by urban/rural mix PHUs (94.5 per 100,000 population), while urban PHUs reported the lowest rate (49.1 per 100,000 population). See Fig. 4 (a) for an overview of crude rates and absolute numbers of OAT prescribers. However, these figures may not accurately reflect the true distribution of prescribers, as individual providers can be counted in multiple regions.

The rate of OAT prescribers varied significantly across rurality as assessed by omnibus test (Appendix A, Table 5). Urban PHUs had significantly lower rates of OAT prescribers compared to rural and sparsely populated PHUs (urban vs. rural: *p* < 0.0001 and urban vs. sparsely populated: *p* < 0.0001). Additionally, urban/rural mix PHUs had significantly lower rates compared to rural and sparsely populated PHUs (urban/rural mix vs. rural: *p* = 0.0063 and urban/rural mix vs. sparsely populated PHUs *p* < 0.0001). Furthermore, sparsely populated PHUs had significantly higher rates compared to rural PHUs (sparsely populated vs. rural: *p* < 0.0001).

##### OAT engagement

Between 2022 and 2023, the average number of OAT clients was 67,074 with a crude rate of 444.4 per 100,000 population for all of Ontario. Sparsely populated PHUs reported the highest average rate of OAT engagement (2000.4 OAT clients per 100,000 population). Rural PHUs also reported higher-than-average rates (697.8 per 100,000 population), followed by urban/rural mix PHUs (491.3 per 100,000 population), while urban PHUs reported the lowest rate (220.7 per 100,000 population). See Fig. 4 (b) for an overview of the crude rates and number of OAT clients in Ontario.

OAT engagement varied significantly across rurality as assessed by omnibus test (Appendix A, Table 6). Urban PHUs had significantly lower rates of OAT engagement compared to rural and urban/rural mix PHUs (urban vs. rural: *p* < 0.0001 and urban/rural mix: *p* = 0.049). There were no significant differences found in the rate of OAT engagement between other geographic regions.

#### Distribution of harm reduction supplies

##### Naloxone distribution

**Fig. 5:**
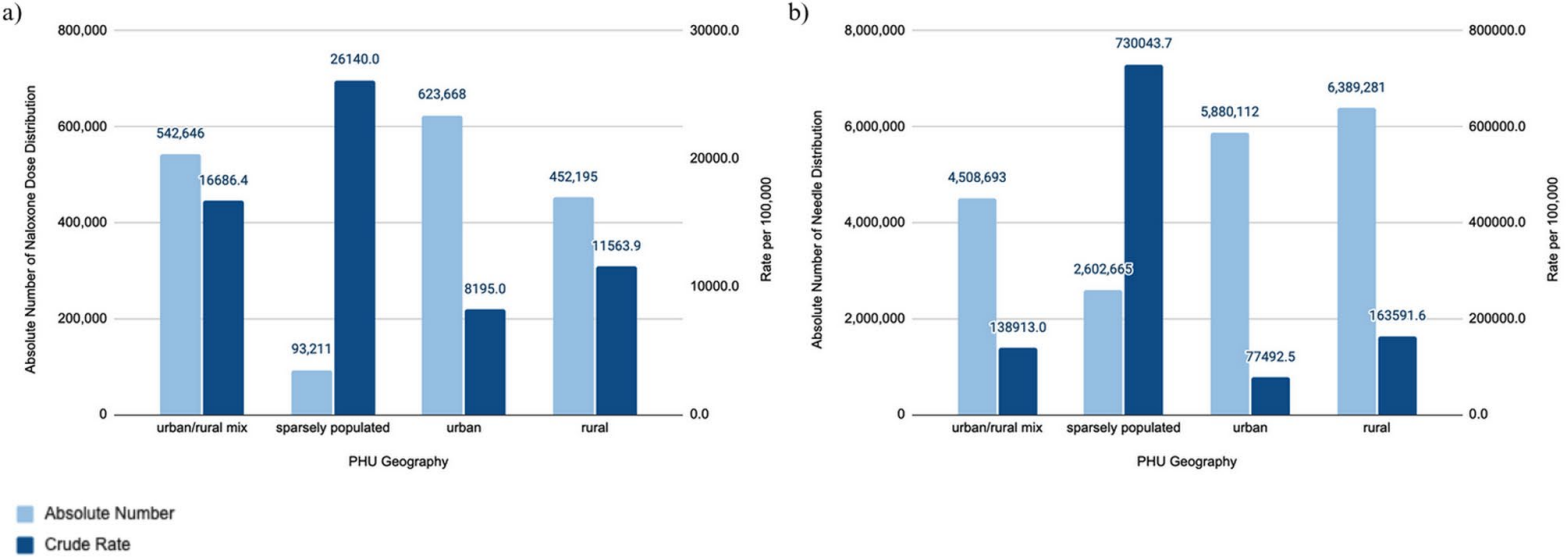
The 2022-2023 average number and rate of naloxone dose and needle distribution in urban, urban/rural mix, rural, and sparsely populated regions in Ontario. The following indicators are presented: (**a**) Naloxone Dose Distribution, and (**b**) Needle Distribution

Between 2022 and 2023, the average number of naloxone doses distributed was 1,711,720 with a crude rate of 11,316 per 100,000 population for all of Ontario. Sparsely populated PHUs reported the highest average rate of naloxone dose distribution (26,140 naloxone doses per 100,000 population). Urban/rural mix PHUs also reported higher-than-average rates (16,686 per 100,000 population), followed by rural PHUs (11,564 per 100,000 population), while urban PHUs reported the lowest rate (8,195 per 100,000 population). See Fig. 5 (a) for an overview of the crude rates and number of naloxone kits distributed in Ontario.

Naloxone distribution did not vary significantly across rurality as assessed by omnibus test (Appendix A, Table 10).

##### Needle distribution

Between 2022 and 2023, the average number of needles distributed was 19,380,751 with a crude rate of 128,381 per 100,000 population for all of Ontario. Sparsely populated PHUs reported the highest average rate of needles distributed (730,044 needles per 100,000 population). Rural PHUs also reported higher-than-average rates (163,592 per 100,000 population), followed by urban/rural mix PHUs (138,913 per 100,000 population), while urban PHUs reported the lowest rate (77,492 per 100,000 population). See Fig. 5 (b) for an overview of the crude rates and number of naloxone kits distributed in Ontario.

Needle distribution varied significantly across rurality as assessed by omnibus test (Appendix A, Table 11). Urban PHUs had significantly lower rates of needle distribution compared to sparsely populated PHUs (urban vs. sparsely populated: *p* = 0.0081). There were no significant differences found in the rate of needle distribution between other geographic regions.

## Discussion

This study presents data on the absolute number and crude rates of various opioid-related indicators, highlighting significant geographic disparities in opioid-related harms, opioid-inclusive service provision, and the distribution of harm reduction supplies across Ontario. While opioid-related harms affect all PHUs, sparsely populated and rural PHUs face a disproportionate impact, with consistently higher rates of opioid-related ED visits, hospitalizations, and deaths. At the same time, while some sparsely populated PHUs report higher rates of service availability, particularly opioid-inclusive treatment programs, the persistently high rates of opioid-related harms may suggest a disconnect between service presence and actual accessibility, appropriateness, or impact. Finally, sparsely populated PHUs report the highest rate of the distribution of harm reduction supplies, highlighting that harm reduction supplies are commonly utilized, when available, in these regions.

### Interpreting opioid-related harms and service provision: a complete picture

Our findings highlight the need to present all opioid-related data, including service provision and distribution of harm reduction supplies in both absolute numbers and crude rates. When data are presented solely in absolute terms, opioid-related harms (ED visits, hospitalizations, and deaths) appear most concentrated in urban areas, however, when considering crude rates, the burden of opioid-related harms is disproportionately higher in sparsely populated and rural PHUs. A similar pattern emerges when looking at the distribution of harm reduction supplies, specifically needles, where absolute numbers reveal that needle distribution in urban PHUs was highest, whereas crude rates reveal that sparsely populated PHUs far surpass urban PHUs.

The importance of presenting both crude rates and absolute numbers is also evident when examining opioid-inclusive services. For instance, while sparsely populated PHUs report the highest crude rate of harm reduction services, they are served by only six harm reduction programs in total. In contrast, urban PHUs have 45 harm reduction programs, significantly more in absolute terms, but report a lower rate of service provision. These discrepancies are likely due to different population sizes, where urban PHUs have substantially larger populations rather than a higher per capita burden of opioid-related harms [15]. For example, in 2024, urban PHUs had a combined population size of nearly 8 million, while rural PHUs had approximately four million residents, and sparsely populated PHUs collectively had a population of just 357,156 residents [22]. This contrast illustrates how relying on either measure alone can misrepresent the actual availability and accessibility of services in a given region. For policymakers, community leaders, decision-makers, and service providers, a comprehensive understanding of both metrics ensures informed, data-driven decisions about where resources and interventions are most urgently needed.

### Geographic disparities in opioid-related harms and opioid-inclusive services

Our findings show that the rates of opioid-related harms in both rural and sparsely populated PHUs significantly exceed those in urban regions, which contrasts the high absolute numbers of opioid-related harms in urban PHUs, which is consistent with research from Canada and the United States highlighting the disproportionate burden of opioid-related harms in rural and sparsely populated areas [7, 15]. For example, rates of opioid-related ED visits and hospitalizations in sparsely populated PHUs were over 2.5 times higher than those in urban PHUs, while opioid-related deaths were 3.5 times higher. Further, rural PHUs reported rates approximately double that of urban PHUs for every opioid-related harm indicator. Notably, both sparsely populated and rural PHUs reported rates above the provincial average for every opioid-related harm indicator, whereas urban PHUs consistently fell below the provincial average. Ongoing surveillance data from 2024 further reinforce these findings, showing that sparsely populated PHUs continue to experience the highest rates of opioid-related harms, while urban PHUs consistently report the lowest rates [22]. For example, preliminary 2024 data show that sparsely populated PHUs reported an average crude rate of 45.4 ED visits per 100,000 population (*n* = 162), compared to urban PHUs which reported an average crude rate of 15.8 ED visits per 100,000 population (*n* = 1237) [22]. These disparities highlight a heightened risk of severe health outcomes within sparsely populated and rural PHUs.

Building on these persistent disparities in opioid-related harms, this study highlights a critical misalignment between the provision of opioid-inclusive services and the needs of local communities, particularly regarding harm reduction programs. While sparsely populated PHUs report the highest crude rates of harm reduction programs (more than double both the provincial average and that of urban PHUs), these rates do not reflect the true accessibility of services within these regions. In reality, 50% of all harm reduction programs in Ontario are concentrated in urban PHUs, while sparsely populated PHUs, despite experiencing the highest rates of opioid-related harms, account for only 7% of these programs, all of which are concentrated within one PHU [17]. 

Expanding access to harm reduction supplies in rural and sparsely populated PHUs is essential to meet growing demand. For instance, needle distribution rates in sparsely populated PHUs are approximately 9.5 times higher than in urban PHUs and 5.5 times higher than the provincial average. Naloxone distribution rates are similarly elevated, over three times higher than in urban PHUs and double the provincial average. These figures indicate substantial reliance on harm reduction supplies in these regions. However, despite higher rates of distribution, service gaps exist. An interactive map by the *Ontario Harm Reduction Distribution Program (OHRDP)* illustrates that many communities across Northern Ontario, including numerous Indigenous communities, lack any local programs distributing harm reduction supplies [31]. This suggests that while distribution rates are high, they may be centralized within a small number of Northern communities, leaving large areas without adequate access. Given the limited number of harm reduction programs and the geographic isolation of many communities within sparsely populated PHUs, current service availability is unlikely to meet the full extent of each communities need. These findings emphasize the importance of not only scaling up harm reduction programs in these regions but also expanding the decentralized distribution of harm reduction supplies to improve equitable access across sparsely populated PHUs.

Importantly, proximity to harm reduction services plays a crucial role in shaping service utilization and health outcomes. Individuals residing in rural and sparsely populated PHUs often face significant geographic barriers to accessing harm reduction services, which can lead to increased engagement in high-risk behaviors, such as needle sharing and reuse if these supplies are not closely accessible [32], and higher rates of overdose mortality [33, 34]. For example, research from Toronto found that half of PWUD were willing to travel up to 10 blocks to access a supervised injection facility, while in Ottawa, 40% of PWUD shared they would walk no more than 10 min to access such a service [35]. These findings underscore the importance of close and convenient access to opioid-inclusive services, particularly in regions where distance can be a critical barrier to care.

While urban PHUs maintain relatively high absolute numbers across all service types (treatment, support, counselling, and harm reduction services), service provision in sparsely populated and rural PHUs appears more concentrated in specific areas, particularly opioid-inclusive treatment services. For example, rural PHUs reported the highest absolute number of treatment programs in the province, while harm reduction services remain disproportionately lacking. This distinction is further reflected in the distribution of OAT prescribers. Sparsely populated PHUs reported OAT prescriber rates over three times the provincial average and six times that of urban PHUs, while rural PHUs reported rates 1.5 times the provincial average and three times that of urban PHUs. These elevated rates of OAT prescribers in rural and sparsely populated PHUs may reflect both a lack of alternative opioid-inclusive services (such as harm reduction or counselling) and an overreliance on OAT as one of the few accessible treatment options. In contrast, urban PHUs offer a more diverse range of opioid-inclusive services, including withdrawal management programs, outpatient addiction services, and CTS/SCS, allowing for a broader range of treatment pathways beyond OAT alone.

### The need for tailored interventions: potential barriers to accessibility in rural and sparsely populated PHUs

Despite the reported availability of opioid-inclusive services, with the exception of harm reduction programs, opioid-related harms remain highest in rural and sparsely populated PHUs, suggesting that structural and systemic barriers play a significant role in driving these disparities. Geographic isolation, centralized services, long emergency response times, and limited transportation options can delay life-saving interventions, such as naloxone administration or access to addiction treatment, even when services technically exist [6, 15, 36, 37]. In Northern Ontario, for example, the four sparsely populated PHUs cover a vast 705,235 km, making service access particularly difficult [18]. Seasonal weather conditions, especially during winter, and the absence of public transportation further exacerbates these barriers, with some communities only accessible by air travel [8, 18]. While fly-in providers do offer services like OAT in certain remote communities, these visits require extensive coordination and are infrequent. In some cases, a temporary physician may fly in once a month to initiate new OAT clients, with follow-up care relying heavily on virtual services; a model that remains less accessible than the continuous, in-person care available in urban settings [38]. 

Beyond geographic barriers, social determinants of health, such as poverty, housing instability, unemployment, and experiences of racism and stigma, disproportionately affect rural and sparsely populated PHUs, increasing vulnerability to opioid use and related harms in these regions [8, 39, 40]. These challenges are further compounded for Indigenous populations, who face additional systemic inequities rooted in the ongoing impacts of colonization, intergenerational trauma, and structural discrimination within healthcare, housing and social support systems, resulting in further barriers to accessing culturally safe and appropriate care [41, 42]. Rural and sparsely populated areas also report higher rates of poverty and job loss, both of which are known risk factors for opioid-related harms [6]. Stigma is particularly critical to address in these regions, where concerns about privacy and confidentiality often discourage individuals from seeking treatment or accessing harm reduction services and supplies [8]. In smaller communities, fear of being recognized by others frequently prevents people from engaging with available supports [8, 43]. Ultimately, these socioeconomic factors, combined with the pervasive stigma associated with drug use create significant barriers to care, reinforce isolation and contribute to cycles of addiction and harm, even when services are present [8, 39, 41, 42]. 

Additionally, the limited harm reduction services in rural and sparsely populated PHUs has critical implications for overdose prevention and public health, particularly given the strong evidence supporting their effectiveness in reducing opioid-related harms by preventing overdoses and facilitating timely medical intervention [15, 40, 44–47]. For example, Thunder Bay’s CTS/SCS which integrated an SSP and drug checking services, demonstrated positive health outcomes [47]. Between October 2022 and March 2024, the site reported a 92% reduction in overdoses among clients, with no fatal overdose, and 50% of SSP clients reported fewer ED visits since enrollment, largely attributed to improved access to primary care and wraparound supports [46]. Despite these successes, this site, along with nine other CTS/SCS in Ontario, have closed as of April 1, 2025, following policy changes by the Ontario government [48]. Additionally, funding for SSPs has expired, and future applications for CTS/SCS and SSPs must now receive provincial approval prior to seeking future funding, further threatening the already limited availability of harm reduction programs, particularly in regions where services are most needed [49]. 

### Addressing geographic disparities

Given that opioid-related harms remain highest in rural and sparsely populated areas despite reported service provision, addressing these disparities requires more than simply increasing the number of available services. Many individuals face barriers to engaging in formal treatment due to personal circumstances, previous negative experiences with healthcare providers, or rigid program requirements such as abstinence from polysubstance use, frequent urine testing, or limited treatment options [17, 38]. OAT programs must be culturally safe and delivered by trained professionals who address stigma and provide judgement-free care [50]. This is especially important in Northern and sparsely populated PHUs, where Indigenous populations represent a large proportion of residents, and where Indigenous healing practices should be integrated in models of care [17, 41]. 

Expanding telehealth and virtual services represents an important strategy to bridge service gaps in geographically isolated and underserved communities. Telehealth platforms have demonstrated success in improving access to OAT, reducing geographic barriers, and achieving health outcomes comparable to in-person care for individuals with OUD [17, 39, 43, 51]. Virtual overdose monitoring services, where peer workers remotely supervise drug use and can initiate emergency responses if needed, offer additional support but cannot fully replace in-person supervision, particularly in rural and sparsely populated regions where emergency response times may be delayed [37, 52]. 

Mobile units and secondary distribution programs (or satellite sites) should also be expanded to improve access to harm reduction programs and supplies, especially in communities without fixed-site services. Research has shown that mobile units can be highly effective in rural settings. For example, an evaluation of a mobile CTS/SCS in rural British Columbia found that over 90% of clients reported positive experiences, citing improved access to safer consumption spaces and harm reduction resources [53]. Mobile programs that partner with local organizations and offer wraparound supports, such as wound care, social services, and referrals can further enhance their reach and impact within underserved communities [54]. However, operational challenges remain, including weather-related service interruptions, transportation barriers, and difficulties maintaining appropriate conditions within mobile units, particularly in harsh winter climates [53]. Secondary distribution programs have also proven successful in expanding access to harm reduction supplies in urban areas, yet limited research exists on their implementation in non-urban regions where they may offer particular benefit for remote and dispersed populations [35]. As such, while mobile and telehealth services are essential tools for improving service reach in rural and sparsely populated PHUs, they should complement, not replace fixed-site programs to ensure sustainable, reliable and equitable access to care.

Additionally, the voluntary merging of some rural and sparsely populated PHUs in Ontario presents both opportunities and challenges [55]. While mergers may improve system coordination and reduce administrative costs, concerns arise when mergers require a minimum population base of 500,000, a threshold difficult to achieve in low-density regions without combining multiple geographically vast areas [56]. Such mergers risk expanding service areas so significantly that access to care, especially harm reduction and emergency services, could be further compromised. Centralizing services in larger hubs may force individuals in remote communities to travel even greater distances for care, exacerbating existing barriers. This has led some PHUs, such as Northwestern and Thunder Bay, to opt out of merging, citing concerns that expanded service areas would hinder rather than improve service provision [57]. Moving forward, any merger or restructuring efforts must be carefully designed to prioritize local access and address the unique needs of rural and sparsely populated PHUs. Without such considerations, PHU mergers risk deepening existing inequities in opioid-related harms and service provision, further disadvantaging regions already disproportionately impacted by the overdose crisis.

### Limitations

This study has several limitations. Firstly, discrepancies exist across databases reporting opioid-related health indicators, such as ED visits, hospitalizations, and deaths. Individual PHU websites often report higher rates than PHO’s “*Substance Use and Harms”* tool, yet PHO was selected as the primary data source due to its comprehensiveness and consistency, as individual PHU websites do not always provide quarterly data. For example, PHO’s “*Substance Use and Harms”* tool reported 1,263 opioid-related ED visits in Ottawa Public Health in 2023, while *Ottawa’s Overdose Overview* reported 1,338 ED visits in 2023 [58]. 

Secondly, no existing database classifies Ontario’s PHUs into urban, urban/rural mix, rural, and sparsely populated categories, necessitating our own classification based on Statistics Canada’s Health Region Peer Groups. While this approach offers a structured framework, it may not fully capture the nuances of opioid-related harms and service needs across the province.

Thirdly, there are gaps between reported and actual service availability. ConnexOntario, the most comprehensive service directory, relies on voluntary submissions, meaning some programs may not be listed. Service details are verified only once per year, potentially leading to outdated or incomplete data. For example, ConnexOntario, identified 22 harm reduction programs, whereas cross-referencing with Health Canada’s website identified 87, though this count only included CTS/SCS, SSPs, drug checking programs, and mobile outreach services. Additionally, ConnexOntario categorizes programs by their primary service type, potentially underrepresenting services that offer multiple forms of support.

Fourth, while this study draws on opioid-related harm data and harm reduction supply distribution data from 2022 to 2023, opioid-inclusive service data reflect the most recent information available as of January 2025. The use of these datasets remains appropriate, as trends in opioid-related harms have remained consistent over time, supporting the validity of the findings and their relevance to current service needs.

Lastly, OAT prescriber counts are based on client location rather than prescriber location, as reported by ODPRN. As a result, OAT prescribers may be counted in multiple PHUs, potentially inflating OAT prescriber numbers when aggregated across regions.

## Conclusion

The overdose crisis in Ontario is not limited to urban centers, it is an escalating public health emergency in rural and sparsely populated PHUs. While urban PHUs report the highest absolute numbers of opioid-related harms, crude rates reveal a more troubling reality; sparsely populated and rural PHUs experience the highest burden of opioid-related ED visits, hospitalizations, and deaths. Further, the prevalence of OAT prescribers and utilization, and needle and naloxone distribution, in conjunction with a lack of harm reduction services in rural and sparsely populated PHUs–evidenced by the centralization and low absolute numbers of harm reduction programs–demonstrate the need for alternative opioid-inclusive services, such as SSPs and CTS/SCS; all of which have been significantly scaled back in Ontario due to forced closures and funding expirations. As such, relying solely on either absolute numbers or crude rates in data may mislead decision-makers into underestimating the crisis in non-urban areas, resulting in disproportionate funding and resource allocation in urban areas, while sparsely populated and rural regions continue to experience worsening opioid-related harms with inadequate support. Understanding the geographic variations in opioid-related harms and service provision is critical in providing insights into the disparities faced by rural and non-urban communities. Without targeted service interventions for rural and sparsely populated communities, the disparities in opioid-related health outcomes will continue to grow, as services designed for urban communities are not successful in more geographically isolated areas, leaving the most vulnerable populations at an even greater risk of preventable overdose and death. Addressing these disparities is key to reducing opioid-related mortality and ensuring equitable access to life-saving services across Ontario. Furthermore, this research can lay the groundwork for a more coordinated provincial response, ensuring that all individuals, regardless of geography, receive the care and support they need to combat the overdose crisis effectively.

## Supplementary Information

Supplementary Material 1.

## Data Availability

The datasets generated and/or analysed during the current study are available in the following repositories: OAT Clients and Prescribers: [https://odprn.ca/ontario-opioid-indicator-tool/oat/]Opioid-Related Health Indicators and Population: [https://www.publichealthontario.ca/en/data-and-analysis/substance-use/substance-use-harms-tool]. Summaries of the datasets used are included in this published article in the form of graphs.

## Abbreviations

alPHa: Association of Local Public Health Agency
ANOVA: Analysis of Variance
ASH: Addictions Supportive Housing
CIHR: Canadian Institute of Health Research
CTS/SCS: Consumption Treatment Services/Supervised Consumption Sites
ED: Emergency Department
GTA: Greater Toronto Area
HSD: Honestly Significant Difference
NMS: Narcotics Monitoring System
PHO: Public Health Ontario
PHU: Public Health Unit
OAT: Opioid Agonist Treatment
ODB: Ontario Drug Benefit
OHIP: Ontario Health Insurance Plan
ODPRN: Ontario Drug Policy Research Network
ONP: Ontario Naloxone Program
ONPP: Ontario Naloxone Program for Pharmacies
OUD: Opioid Use Disorder
RAAM: Rapid Access Addiction Medicine
SROM: Slow-Release Oral Morphine
SSP: Safer Supply Program
