# Supplementary material for: Mapping the overdose crisis in Ontario: geographic disparities in opioid-related harms and services
Source: BMC Public Health. 2025 Nov 13;25:3935. doi: 10.1186/s12889-025-25103-y (PMC12616965; doi:10.1186/s12889-025-25103-y)
Supplement: Supplementary file 1 — Supplementary Material 1. [file 12889_2025_25103_MOESM1_ESM.docx]

**Appendix A: Statistical Analysis Results**

**Table 1. Rate of Emergency Department Visits (2022-2023)**

| **Global One-Way ANOVA** | | | | | |
| --- | --- | --- | --- | --- | --- |
| **Source** | **Df** | **Sum Sq** | **Mean Sq** | **F value** | **Pr(>F)** |
| Rurality | 3 | 24524.56 | 8174.854 | 3.294067 | 0.033877 |
| Residuals | 30 | 74450.72 | 2481.691 | -- | -- |
| **Tukey HSD Pairwise Test** | | | | | |
| **Pairwise Comparison** | | **Diff** | **Lwr 95% CI** | **Upr 95% CI** | **p adj** |
| Sparsely Populated | Urban/Rural Mix | 61.4967 | -25.9403 | 148.9336 | 0.2444 |
| Urban | Urban/Rural Mix | -36.1107 | -118.134 | 45.9125 | 0.6334 |
| Rural | Urban/Rural Mix | 23.7867 | -39.6467 | 87.2201 | 0.7394 |
| Urban | Sparsely Populated | -97.6073 | -188.474 | -6.7402 | 0.0316 |
| Rural | Sparsely Populated | -37.71 | -112.227 | 36.8074 | 0.5236 |
| Rural | Urban | 59.8973 | -8.1865 | 127.9812 | 0.1004 |

**Table 2. Rate of Hospitalizations (2022-2023)**

| **Global One-Way ANOVA** | | | | | |
| --- | --- | --- | --- | --- | --- |
| **Source** | **Df** | **Sum Sq** | **Mean Sq** | **F value** | **Pr(>F)** |
| Rurality | 3 | 718.468 | 239.4893 | 6.402022 | 0.001751 |
| Residuals | 30 | 1122.252 | 37.40839 | -- | -- |
| **Tukey HSD Pairwise Test** | | | | | |
| **Pairwise Comparison** | | **Diff** | **Lwr 95% CI** | **Upr 95% CI** | **p adj** |
| Rural | Sparsely Populated | -5.925 | -15.0739 | 3.2238 | 0.3115 |
| Rural | Urban | 10.9651 | 2.6061 | 19.3242 | 0.0064 |
| Rural | Urban/Rural Mix | 2.6353 | -5.1528 | 10.4233 | 0.7944 |
| Urban | Sparsely Populated | -16.8902 | -28.0464 | -5.734 | 0.0015 |
| Sparsely Populated | Urban/Rural Mix | 8.5603 | -2.1748 | 19.2954 | 0.1554 |
| Urban | Urban/Rural Mix | -8.3299 | -18.4003 | 1.7405 | 0.1332 |

**Table 3. Rate of Deaths (2022-2023)**

| **Global One-Way ANOVA** | | | | | |
| --- | --- | --- | --- | --- | --- |
| **Source** | **Df** | **Sum Sq** | **Mean Sq** | **F value** | **Pr(>F)** |
| Rurality | **3** | **1830.624** | **610.2079** | **5.347363** | **0.00452** |
| Residuals | **30** | **3423.414** | **114.1138** | **--** | **--** |
| **Tukey HSD Pairwise Test** | | | | | |
| **Pairwise Comparison** | | **Diff** | **Lwr 95% CI** | **Upr 95% CI** | **p adj** |
| Sparsely Populated | Urban/Rural Mix | 20.1039 | 1.3544 | 38.8534 | 0.032 |
| Urban | Urban/Rural Mix | -7.8266 | -25.4152 | 9.762 | 0.6255 |
| Rural | Urban/Rural Mix | 4.346 | -9.2563 | 17.9483 | 0.8208 |
| Urban | Sparsely Populated | -27.9305 | -47.4156 | -8.4454 | 0.0027 |
| Rural | Sparsely Populated | -15.7579 | -31.737 | 0.2212 | 0.0544 |
| Rural | Urban | 12.1726 | -2.427 | 26.7721 | 0.1287 |

**Table 4. Rate of OAT Engagement (2022-2023)**

| **Welch’s One-Way ANOVA** | | | | | |
| --- | --- | --- | --- | --- | --- |
| **Source** | **Df Numerator** | **Df Denominator** | | **F value** | **Pr(>F)** |
| Rurality | 3 | 8.9371 | | 24.468 | 0.0001 |
| **Games-Howell Pairwise Test** | | | | | |
| **Pairwise Comparison** | | **Diff** | **Lwr 95% CI** | **Upr 95% CI** | **p adj** |
| Rural | Sparsely Populated | 1020.409 | -795.842 | 2836.66 | 0.208 |
| Rural | Urban | -510.516 | -696.127 | -324.905 | <0.0001 |
| Rural | Urban/Rural Mix | -235.108 | -524.806 | 54.5905 | 0.13 |
| Sparsely Populated | Urban | -1530.93 | -3384.83 | 322.977 | 0.082 |
| Sparsely Populated | Urban/Rural Mix | -1255.52 | -3057.5 | 546.4645 | 0.129 |
| Urban | Urban/Rural Mix | 275.4083 | 1.0357 | 549.781 | 0.049 |

**Table 5. Rate of OAT Prescriber (2022-2023)**

| **Global One-Way ANOVA** | | | | | |
| --- | --- | --- | --- | --- | --- |
| **Source** | **Df** | **Sum Sq** | **Mean Sq** | **F value** | **Pr(>F)** |
| Rurality | 3 | 196363.1 | 65454.37 | 37.87996 | <0.0001 |
| Residuals | 30 | 51838.26 | 1727.942 | **--** | **--** |
| **Tukey HSD Pairwise Test** | | | | | |
| **Pairwise Comparison** | | **Diff** | **Lwr 95% CI** | **Upr 95% CI** | **p adj** |
| Sparsely Populated | Urban/Rural Mix | 230.8737 | 157.9135 | 303.8338 | <0.0001 |
| Urban | Urban/Rural Mix | -46.0621 | -114.505 | 22.3806 | 0.2796 |
| Rural | Urban/Rural Mix | 69.5809 | 16.6501 | 122.5118 | 0.0063 |
| Urban | Sparsely Populated | -276.936 | -352.758 | -201.113 | <0.0001 |
| Rural | Sparsely Populated | -161.293 | -223.472 | -99.1131 | <0.0001 |
| Sparsely Populated | Urban/Rural Mix | 230.8737 | 157.9135 | 303.8338 | <0.0001 |

**Table 6. Rate of Treatment Services (2024)**

| **Kruskal-Wallis Test** | | | | | | | |
| --- | --- | --- | --- | --- | --- | --- | --- |
| **Source** | **Df** | | **H Statistic** | | | **P Value** | |
| Rurality | **3** | | **16.90214** | | | **0.00074** | |
| **Dunn’s Pairwise Test (with Benjamini-Hochberg Correction)** | | | | | | | |
| **Pairwise Comparison** | | **Diff** | | **Lwr 95% CI** | **Upr 95% CI** | | **p adj** |
| Urban/Rural Mix | **Sparsely Populated** | **-20.0781** | | **-28.3381** | **-11.818** | | **0.0136** |
| Urban/Rural Mix | **Urban** | **2.0116** | | **0.3034** | **3.7197** | | **0.1963** |
| Urban/Rural Mix | **Rural** | **-1.9778** | | **-4.0939** | **0.1382** | | **0.3206** |
| Sparsely Populated | **Urban** | **22.0896** | | **13.8736** | **30.3057** | | **0.0004** |
| Sparsely Populated | **Rural** | **18.1002** | | **9.7898** | **26.4106** | | **0.0194** |
| Urban | **Rural** | **-3.9894** | | **-5.9264** | **-2.0525** | | **0.0185** |

**Table 7. Rate of Support Services (2024)**

| **Kruskal-Wallis Test** | | | |
| --- | --- | --- | --- |
| **Source** | **Df** | **H Statistic** | **P Value** |
| Rurality | **3** | **5.424903** | **0.1432** |

**Table 8. Rate of HR Services (2024)**

| **Kruskal-Wallis Test** | | | |
| --- | --- | --- | --- |
| **Source** | **Df** | **H Statistic** | **P Value** |
| **Rurality** | **3** | **4.417776** | **0.219743** |

**Table 9. Rate of Counselling Services (2024)**

| **Kruskal-Wallis Test** | | | | | | | |
| --- | --- | --- | --- | --- | --- | --- | --- |
| **Source** | **Df** | | **H Statistic** | | | **P Value** | |
| Rurality | 3 | | 19.21439 | | | 0.000247 | |
| **Dunn’s Pairwise Test (with Benjamini-Hochberg Correction)** | | | | | | | |
| **Pairwise Comparison** | | **Diff** | | **Lwr 95% CI** | **Upr 95% CI** | | **p adj** |
| Urban/Rural Mix | Sparsely Populated | -30.0037 | | -45.6662 | -14.3411 | | 0.0018 |
| Urban/Rural Mix | Urban | 1.2327 | | -0.8665 | 3.332 | | 0.588 |
| Urban/Rural Mix | Rural | -4.9302 | | -7.7124 | -2.148 | | 0.0366 |
| Sparsely Populated | Urban | 31.2364 | | 15.5683 | 46.9045 | | 0.0009 |
| Sparsely Populated | Rural | 25.0735 | | 9.2994 | 40.8476 | | 0.0366 |
| Urban | Rural | -6.1629 | | -8.976 | -3.3499 | | 0.014 |

**Table 10. Naloxone Distributed (2022-2023)**

| **Kruskal-Wallis Test** | | | |
| --- | --- | --- | --- |
| **Source** | **Df** | **H Statistic** | **P Value** |
| Rurality | 3 | 5.810721 | 0.121191 |

**Table 11. Needles Distributed (2022-2023)**

| **Kruskal-Wallis Test** | | | | | | | |
| --- | --- | --- | --- | --- | --- | --- | --- |
| **Source** | **Df** | | **H Statistic** | | | **P Value** | |
| Rurality | 3 | | 10.33845 | | | 0.015898 | |
| **Dunn’s Pairwise Test (with Benjamini-Hochberg Correction)** | | | | | | | |
| **Pairwise Comparison** | | **Diff** | | **Lwr 95% CI** | **Upr 95% CI** | | **p adj** |
| Urban/Rural Mix | Sparsely Populated | -577706.2757 | | -1304591.828 | 149179.277 | | 0.0761 |
| Urban/Rural Mix | Urban | 77462.3193 | | -21309.1775 | 176233.8161 | | 0.2172 |
| Urban/Rural Mix | Rural | -17938.9585 | | -108344.085 | 72466.1681 | | 0.8626 |
| Sparsely Populated | Urban | 655168.595 | | -70156.1297 | 1380493.32 | | 0.0081 |
| Sparsely Populated | Rural | 559767.3172 | | -164465.5421 | 1284000.177 | | 0.0667 |
| Urban | Rural | -95401.2777 | | -172254.6212 | -18547.9343 | | 0.1144 |
